# Dietary Supplementation with Selenium and Coenzyme Q_10_ Prevents Increase in Plasma D-Dimer While Lowering Cardiovascular Mortality in an Elderly Swedish Population

**DOI:** 10.3390/nu13041344

**Published:** 2021-04-17

**Authors:** Urban Alehagen, Jan Aaseth, Tomas L. Lindahl, Anders Larsson, Jan Alexander

**Affiliations:** 1 Division of Cardiovascular Medicine, Department of Health, Medicine and Caring Sciences, Linköping University, SE-581 85 Linköping, Sweden; 2 Research Department, Innlandet Hospital Trust, N-2381 Brumunddal, Norway; jaol-aas@online.no; 3 Division of Clinical Chemistry and Pharmacology, Department of Biomedical and Clinical Sciences, Linköping University, SE-581 85 Linköping, Sweden; tomas.lindahl@liu.se; 4 Department of Medical Sciences, Uppsala University, SE-751 85 Uppsala, Sweden; anders.larsson@medsci.uu.se; 5 Norwegian Institute of Public Health, N-0403 Oslo, Norway; Jan.Alexander@fhi.no

**Keywords:** D-dimer, intervention, elderly, cardiovascular mortality, selenium, coenzyme Q_10_

## Abstract

A low intake of selenium is associated with increased cardiovascular mortality. This could be reduced by supplementation with selenium and coenzyme Q_10_. D-dimer, a fragment of fibrin mirroring fibrinolysis, is a biomarker of thromboembolism, increased inflammation, endothelial dysfunction and is associated with cardiovascular mortality in ischemic heart disease. The objective was to examine the impact of selenium and coenzyme Q_10_ on the level of D-dimer, and its relationship to cardiovascular mortality. D-dimer was measured in 213 individuals at the start and after 48 months of a randomised double-blind placebo-controlled trial with selenium yeast (200 µg/day) and coenzyme Q_10_ (200 mg/day) (*n* = 106) or placebo (*n* = 107). The follow-up time was 4.9 years. All included individuals were low in selenium (mean 67 μg/L, SD 16.8). The differences in D-dimer concentration were evaluated by the use of T-tests, repeated measures of variance and ANCOVA analyses. At the end, a significantly lower D-dimer concentration was observed in the active treatment group in comparison with those on placebo (*p* = 0.006). Although D-dimer values at baseline were weakly associated with high-sensitive CRP, while being more strongly associated with soluble tumour necrosis factor receptor 1 and sP-selectin, controlling for these in the analysis there was an independent effect on D-dimer. In participants with a D-dimer level above median at baseline, the supplementation resulted in significantly lower cardiovascular mortality compared to those on placebo (*p* = 0.014). All results were validated with a persisting significant difference between the two groups. Therefore, supplementation with selenium and coenzyme Q_10_ in a group of elderly low in selenium and coenzyme Q_10_ prevented an increase in D-dimer and reduced the risk of cardiovascular mortality in comparison with the placebo group. The obtained results also illustrate important associations between inflammation, endothelial function and cardiovascular risk.

## 1. Introduction

D-dimer is a fragment of degraded fibrin and reflects the activation of fibrinolysis and thrombosis, but also the activity of peripheral artery disease [1]. It is thus an indicator of the fibrin turnover [2]. The most common indications for use of D-dimer are in the diagnosis of venous thromboembolism [3,4,5,6,7], for the exclusion of pulmonary embolism [8] and in the evaluation of recanalisation of pulmonary emboli after anticoagulation [9]. D-dimer is one of the most commonly used biomarkers in clinical medicine [10]. The assay is mainly based on antibodies against D-dimer [11], and as different antibodies are used in commercial kits, there is some variability in the obtained measurements [12].

After successful electro-conversion, the level of D-dimer is reduced in patients with atrial fibrillation; hence, it is believed that the velocity and turbulence of the blood flow is important for the level of D-dimer as well [13,14,15]. However, the occurrence of emboli in atrial fibrillation as a reason for the increased level of D-dimer cannot be ignored. An increased level of D-dimer has also been associated with increased mortality in patients with heart failure [16] and ischaemic heart disease [17,18]. In patients with myocardial infarction, an association between an increased level of D-dimer and increased risk of mortality after a percutaneous coronary intervention has been reported [19,20]. This relation between D-dimer and mortality risk could be explained by the thrombus area in patients with concomitant pulmonary emboli, but there is also a reported association between the myocardial infarct area and level of D-dimer and mortality risk [21]. Hence, D-dimer also could contribute to prognostic cardiovascular risk information, as has also been reported by Bai et al. [22].

Clinical interpretation of D-dimer-values is complicated by the fact that D-dimer increases with age for patients over 50 years [23]. Furthermore, elevated levels of D-dimer could also be the result of inflammatory activity in the absence of thromboembolism [24]. It has been reported that D-dimer and C-reactive protein (CRP) both provide prognostic information in patients with acute coronary syndromes [25], probably based on the close relation between inflammation and ischaemic heart disease. Recently, a matter of discussion has been the intimate relation between D-dimer and endothelial function, where a dysfunction is an important step in the development of inflammation and structural damage [26]. Therefore, it is of interest to more broadly investigate the inflammatory response by examining the D-dimer response following supplementation with selenium and coenzyme Q_10_. In this context, it is interesting to note that several reports have demonstrated the prognostic properties of D-dimer in patients with COVID-19 disease [22,23,24] emphasising the important association between D-dimer and inflammation and disease prognosis [27,28,29].

Our group has previously reported that in an elderly population with symptoms that could be interpreted as heart failure, D-dimer had prognostic information regarding risk of cardiovascular mortality during a follow-up time of more than five years [30]. Even if exclusion of those with atrial fibrillation or dilated atria as seen on echocardiography, or development of malignant disease during the follow-up time [31,32], the prognostic information remained. Therefore, we assume that an association between D-dimer concentration and cardiovascular disease exists, due for example to increases in atherosclerosis again resulting in a hypercoagulable state [33,34]

Selenium is an essential trace element needed for any human cell in order to fulfil normal cellular functions [35,36]. However, because of soil low in selenium, the dietary intake of selenium is low in European regions, with an estimated intake in European countries of <50 μg/day [37]. In order to obtain an optimal cellular function, the required intake of selenium is at least 75 μg/day of selenium for adult Caucasians [38]. However, to obtain an optimal expression of one of the important selenoproteins; for selenoprotein P, which distributes selenium from the liver to peripheral tissues, a daily intake of 100–150 µg/day of selenium is required [39]. Moreover, in conditions with increased oxidative stress and during inflammation, the need for selenium is increased [40]. These requirements can be met for persons living in regions with an adequate selenium content of the soil, as in the USA. However, in healthy, elderly community-living persons in Sweden, our group has previously reported increased cardiovascular mortality associated with a low intake of selenium [41].

Coenzyme Q_10_ is present in all human cells, where it is active in the mitochondrial respiratory chain, but it is also an important lipid soluble antioxidant. The endogenous production of coenzyme Q_10_ declines with age, and at the age of 80, the endogenous myocardial production of coenzyme Q_10_ is about half that at 20 years of age [42,43].

Cytosolic selenoenzyme thioreductase1 plays a major role in reducing ubiquinone (the oxidised form of coenzyme Q_10_) to ubiquinol, the active, reduced form of coenzyme Q_10_. For an optimal functioning, the cell is both dependent on an adequate supply of coenzyme Q_10_ and synthesis of selenoproteins. An insufficiency in selenium and reduced thioredoxin reductase activity could therefore result in decreased concentrations of active coenzyme Q_10_ (ubiquinol) in the cell. This important relationship between selenium and coenzyme Q_10_ has been known about for a long time [44,45].

Our group has previously observed effects by combined intervention with selenium and coenzyme Q_10_ on several biomarkers for inflammation in a randomised clinical trial on an elderly Swedish population. Thus, the levels of sP-selectin, CRP, osteopontin, osteoprotegerin, soluble tumour necrosis factor receptor 1 (TNFr1) and soluble tumour necrosis factor receptor 2 (TNFr2) were significantly lowered in those receiving active treatment, as compared with those in the placebo group [46,47]. We also observed effects on the biomarker levels of the von Willebrand factor and plasminogen activator inhibitor-1 indicating improved endothelial function in the verum group [48]. With this in mind, we wanted to evaluate if an association between D-dimer levels and supplementation of selenium and coenzyme Q_10_ exists, as D-dimer has also been reported to be associated with endothelial function [26].

Apart from a small study from the former Eastern Germany on 61 patients with myocardial infarction [49], we did not find any other report in the literature using the combined supplementation with selenium and coenzyme Q_10_, which is why the presented results are novel and interesting.

The aim of the present sub-study was to investigate a possible influence of supplementation for four years with selenium and coenzyme Q_10_ on the level of D-dimer, with emphasis on its role in cardiovascular mortality during 4.9 years of follow-up, in an elderly Swedish population.

## 2. Methods

### 2.1. Subjects

From a rural municipality, all individuals living in the age between 69 and 88 years were invited to participate in a study on epidemiology in 1998 (*n* = 1320). Out of those 876 decided to participate in the main project. In 2003, those still alive (*n* = 675) were invited to participate in an intervention project with selenium and coenzyme Q_10_ as a dietary supplement. Due to the fact that some individuals regarded the transportation distance to the Health Center for inclusion as being too long, the number who agreed to participate were 589 individuals. Out of those, 443 individuals in the age 70–88 years agreed to participate in the intervention project. The supplementation consisted of selenium and coenzyme Q_10_, or placebo given over four years, and where blood samples were drawn every 6 months [50]. All participants in the intervention study had a suboptimal pre-intervention serum selenium level, mean 67 μg/L (SD 16.8) (equivalent to an estimated daily intake of 35 μg/day), and this is below what is regarded as an adequate selenium concentration of ≥100 μg/L [51].

In the present sub-analysis on impact on D-dimer, from the group of 443 participants in the intervention, we excluded individuals with conditions known to influence the concentration of D-dimer: atrial fibrillation and/or on treatment with anticoagulants (*n* = 50), participants with malignancies (*n* = 17) or the dimension of the left atrium > 40 mm (*n* = 163). The final population consisted of 213 individuals. Of those, 106 individuals were on active treatment, and 107 individuals were on placebos.

In the main project, the participants received supplementation of 200 mg/day of coenzyme Q_10_ capsules (Bio-Quinon 100 mg B.I.D, Pharma Nord, Vejle, Denmark) and 200 µg/day of organic selenium yeast tablets (SelenoPrecise 100 µg B.I.D, Pharma Nord, Vejle, Denmark) (*n* = 221), or a similar placebo (*n* = 222) over 48 months. After this time, the intervention was finished. The study tablets were taken in addition to any regular medication. All study medications (active drug and placebo) not consumed were returned and counted. One of three experienced cardiologists examined all study participants at the inclusion. Besides a new clinical history, a clinical examination was performed at inclusion and after the study period, including blood pressure, there was an assessment using the New York Heart Association functional class (NYHA class) as well as an electrocardiogram (ECG) and Doppler-echocardiography. Echocardiographic examinations were performed with the participant in the left lateral position. The ejection fraction (EF) readings were categorised into four classes: 30%, 40% and 50% [52,53]. Normal systolic function was defined as EF ≥ 50%, while severely impaired systolic function was defined as EF < 30%. Only the systolic function was evaluated. The inclusion started in January 2003 and finished in February 2010.

The exclusion criteria for the main project were: recent myocardial infarction (within four weeks); planned cardiovascular operative procedure within four weeks; hesitation concerning whether the candidate could decide for him/herself to participate in the study or not, or doubt about whether he/she understood the consequences of participation; serious disease that substantially reduced survival or when it was not expected that the participant could cooperate for the full four-year period; other factors making participation unreasonable, or drug/alcohol abuse [50]. Cardiovascular mortality (CV mortality) was registered for all study participants for a follow-up period of 4.9 years. Information regarding mortality was obtained from the National Board of Health and Welfare in Sweden. It registers all deaths of Swedish citizens based on death certificates or autopsy reports. All patients obtained written informed consent.

Cardiovascular mortality was defined as mortality due to myocardial infarctions, cerebrovascular lesions, fatal cardiac arrythmias, heart failure and aortic aneurysms.

The result of the main study was that the actively treated group showed a significantly increased cardiac systolic function, a reduced concentration of the cardiac peptide N-terminal fragment of B-type natriuretic peptide (NT-proBNP), and significantly reduced cardiovascular mortality [50]. As the result of the main study was surprising, several sub studies were performed. This sub study is one of the different steps in order to obtain better understanding of the mechanisms between supplementation as clinical results.

### 2.2. Biochemical Analyses

All blood samples were collected at start of the study, and after 48 months, and were drawn with the participants resting and in a supine position. Pre-chilled, EDTA vials for plasma were used. The vials were centrifuged at 3000× *g*, +4 °C, and were then frozen at −70 °C. No sample was thawed more than once.

### 2.3. Determination of D-Dimer

Blood was collected in Vacutainer tubes containing 1/10 volume sodium citrate 0.11 mol/L and stored at −70 °C until analysis. The samples were analysed utilising an automated micro-latex D-dimer reagent, MRX-143, from Medirox (Nyköping, Sweden) using ACL Top analyser (Instrumentation Laboratories, Milan, Italy). The precision was good; for a low control at mean concentrations of 0.39 mg/L (*n* = 917) and a high control at 0.96 mg/L (*n* = 526), the total imprecision was 7.3% and 2.9%, respectively.

### 2.4. Statistical Methods

Descriptive data are presented as percentages or mean ± standard deviation (SD). A Student’s unpaired two-sided T-test was used for continuous variables and the chi-square test was used for analysis of one discrete variable. Kaplan–Meier evaluations of all-cause and cardiovascular mortality were made for both the active treatment and placebo groups. The term ‘censored participants’ refers to those still living at the end of the study, or who had died for reasons other than cardiovascular disease. ‘Completed participants’ refers to those who had died due to cardiovascular disease. Repeated measures of variance were used in order to obtain better information on the individual changes in the concentration of the biomarker analysed, compared to group mean values.

In the analysis of covariance (ANCOVA) evaluation, both transformed and non-transformed data were applied, with no significant difference in the results.

In the ANCOVA evaluation, the D-dimer concentration after 48 months was used as an independent variable. In the model, adjustments were made for smoking, hypertension, diabetes, ischaemic heart disease (IHD), NYHA class III, Hb < 120 g/L, statin treatment, P-selectin at inclusion, endostatin at inclusion, soluble tumor necrosis factor receptor 1 (sTNF-r1) at inclusion, sTNF-r2 at inclusion, Growth differentiation factor 15 (GDF-15) at inclusion, D-dimer at inclusion and supplementation with selenium and coenzyme Q_10_.

*p*-values < 0.05 were considered significant, based on a two-sided evaluation. All data were analysed using standard software (Statistica v. 13.2, Dell Inc., Tulsa, OK, USA).

## 3. Results

In Table 1 the baseline characteristics for the active treatment and the placebo groups are presented. It is seen that the two groups are reasonably well balanced with regard to the co-variates analysed.

From the evaluations, 46 out of 213 (22%) had diabetes, 150 out of 213 (70%) had hypertension, 40 out of 213 (19%) had ischaemic heart disease and nine out of 213 (4%) had impaired systolic cardiac function defined as an EF of less than 40%. The population evaluated could be considered as representative for an elderly Swedish population. Upon analysing the association between D-dimer and age at the study start, a significant association was noted (r = 0.20; *p* = 0.003). The mean concentration of D-dimer did not differ between males and females (females: 0.32 mg/L (SD 0.31) vs. males: 0.32 mg/L (SD 0.58); *p* = 0.98). The sub-population studied was followed for 4.9 years from 2003 regarding mortality.

### 3.1. Relation between D-Dimer and Biomarkers for Inflammation at Study Start

As D-dimer has been reported to be associated with biomarkers of inflammation, we examined whether D-dimer was associated with hs-CRP (high sensitive CRP). A weak non-significant association was seen (r = 0.10; *p* = 0.17). However, the size of the association found is as reported by Folsom et al. (r = 0.13) [18]. Stronger associations were seen between D-dimer and soluble tumour necrosis factor (TNF) receptor 1, (r = 0.35; *p* > 0.0001), and soluble TNF receptor 2 (r = 0.24; *p* = 0.01). We also found a significant association with sP-selectin (r = 0.17; *p* = 0.01), another biomarker for inflammation, which is also a marker for platelet activation.

### 3.2. Effect of Supplementation on the Concentration of D-Dimer

At inclusion, there was no significant difference in the mean concentration of D-dimer between the two groups (active: 0.29 mg/L vs. placebo: 0.36 mg/L; *p* = 0.27). However, after 48 months, a significant difference in the concentration of D-dimer between the two groups could be seen (active: 0.22 mg/L vs. 0.34 mg/L; T-value: 2.80; *p* = 0.006).

**Figure 1:**
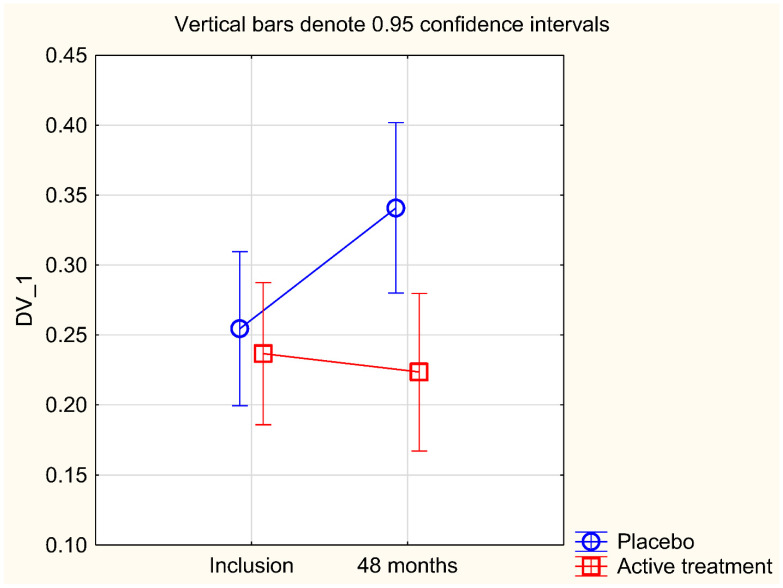
Concentration of D-dimer at the start of the project and after 48 months in the selenium and coenzyme Q_10_ treatment group compared to the placebo group in the study population. Evaluation performed by use of repeated measures of variance methodology. Current effect: (F (1, 111) = 5.11; *p* = 0.026). Vertical bars denote 0.95 confidence intervals. Blue curve: Placebo; Red curve: Active treatment group. Bars indicate ±95% CI.

As a validation of the results obtained, we performed a repeated measures of variance analysis (Figure 1). From this evaluation, the difference between the two groups, active vs. placebo, was still significant (F (1, 111) = 5.11; *p* = 0.026).

As a second step in the validation of the obtained results, an ANCOVA analysis was performed (Table 2).

We found a significantly lower concentration of D-dimer (*p* = 0.002) in those supplemented with selenium and coenzyme Q_10_, also after adjusting for co-variates that might influence the concentration of D-dimer, like the CRP, sP-selectin, TNF-r1, TNf-r2, endostatin and GDF-15, all of which being biomarkers of inflammation.

### 3.3. Effect of Supplementation with Selenium and Coenzyme Q_10_ on Mortality

This sub-study population was followed during a median follow-up period of 4.9 years from 2003. As the study population was relatively small, we chose to evaluate the groups where the risk of mortality was highest. As it has been shown that an increased level of D-dimer increases this risk, we chose to include those with a concentration of D-dimer above the median (0.21 mg/L) at baseline for an evaluation of CV mortality. A significantly lower fraction suffering from CV mortality was seen in those on active treatment, compared with those on placebo (active treatment: one out of 53 vs. placebo: eight out of 52; χ^2^: 6.10; *p* = 0.014). When comparing all-cause mortality in the two groups, the mortality in the placebo group was twice that in the active treatment group, but this difference did not reach statistical significance (active treatment: five out of 53 vs. placebo: 10 out of 52; χ^2^: 2.06; *p* = 0.15). Of note, the groups were small, which probably contributed to the non-significance of the latter difference.

**Figure 2:**
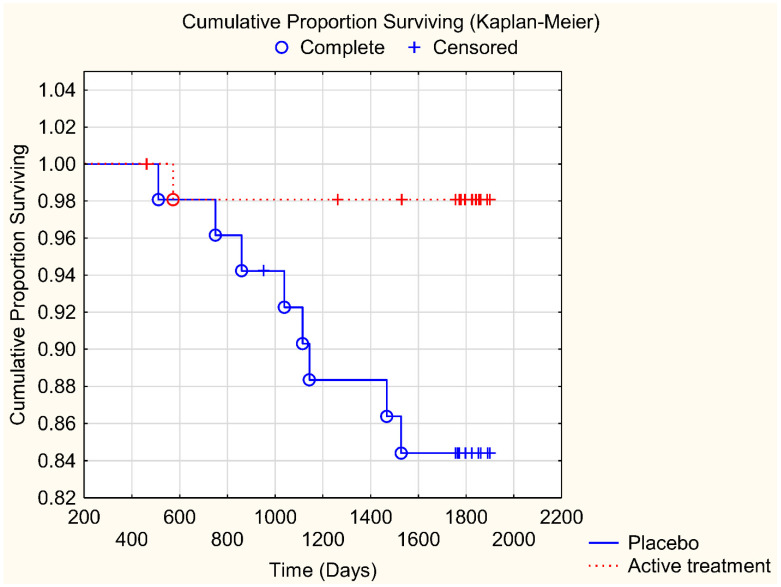
Kaplan–Meier graph illustrating cardiovascular mortality in participants with a D-dimer level above median (0.21 mg/L) and given selenium and coenzyme Q_10_ treatment versus those on placebo during a follow-up period of 4.9 years. Note: Censored participants were those still living at the end of the study period, or who had died for reasons other than cardiovascular disease. Completed participants were those who had died due to cardiovascular disease.

In order to validate the obtained differences in CV mortality between those on active treatment versus those on placebo, a Kaplan–Meier analysis was performed (Figure 2). From that, it could be seen that significantly fewer participants suffered from CV mortality among those who were given active treatment, as compared to those on placebo (z = 2.39; *p* = 0.017).

### 3.4. Impact of Supplementation on D-Dimer Levels in Participants with Hypertension or Ischaemic Heart Disease

**Figure 3:**
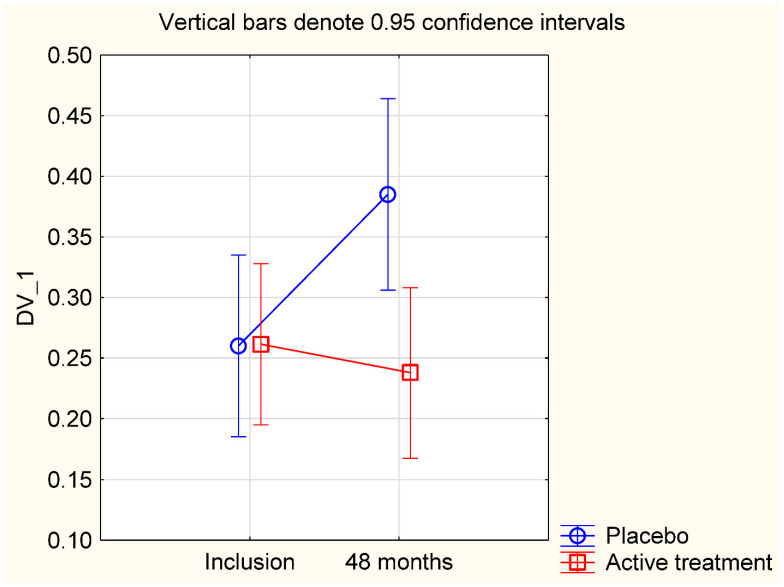
Concentration of D-dimer at the start of the project and after 48 months in the selenium and coenzyme Q_10_ treatment group compared to the placebo group in a sub-group of the study population consisting of participants with hypertension or ischaemic heart disease. Evaluation performed by use of repeated measures of variance methodology. Current effect: (F (1, 75) = 6.23; *p* = 0.015). Vertical bars denote 0.95 confidence intervals. Blue curve: Placebo; Red curve: Active treatment group. Bars indicate ±95% CI.

We conducted a sub-group analysis on participants with hypertension, and/or with ischaemic heart disease, diseases where inflammation is an inseparable part of the picture. In this sub-population, we evaluated the group with a D-dimer concentration above the median (>0.21 mg/L) at baseline. Also in this group, we found a significant difference in impact of the treatment on D-dimer concentration between those on active treatment and those on placebo, when applying the repeated measures of variance methodology (F (1, 75) = 6.23; *p* = 0.015) (Figure 3).

Upon analysing mortality, we found that those on active treatment had a significantly lower CV mortality, compared with those on placebo (active: one out of 46 vs. placebo: six out of 41; χ^2^: 4.55; *p* = 0.033). There was no significant difference in all-cause mortality (active: four out of 46 vs. placebo: eight out of 41: χ^2^: 2.13; *p* = 0.14). However, these groups were small, and consequently the results should be interpreted with caution.

## 4. Discussion

The present evaluations of the effect of supplementation with selenium and coenzyme Q_10_ on the level of D-dimer in an elderly community-living population in Sweden has shown that this treatment prevented an increase in D-dimer levels, as compared with the placebo group in which the levels appeared to increase. Also, in a sub-group analysis of patients with hypertension or ischemic heart disease, a significantly lower concentration of D-dimer as a result of the supplementation was observed. In those with a D-dimer level above the median at baseline, the supplementation resulted in significantly lower cardiovascular mortality compared with those on placebo. Although the studied subjects had a low serum selenium at inclusion (mean 67 μg/L, SD 16.8), which is lower than recommended [51], we consider the studied group to be representative for an elderly Swedish population.

From the main study (referred to as the KiSel-10 project), we have previously disclosed that intervention with selenium and coenzyme Q_10_ caused reduced levels of biomarkers of inflammation [34,35]. As regards this elderly population with a suboptimal selenium status, our group has also observed a significant reducing effect on von Willebrand factor, and plasminogen activator inhibitor-1, by the supplementation with selenium and coenzyme Q_10_ [48]. These latter biomarkers, which are indicators of endothelial function, suggest development of dysfunction in non-supplemented elderly controls. In the present study, we aimed at focusing on D-dimer as an additional biomarker for endothelial dysfunction.

The sub-population included here was selected with the aim of eliminating clinical conditions (atrial fibrillation, increased left atrium size, treatment with anticoagulants and malignancies) known to increase the level of D-dimer. As a result, the sample size was reduced by about 50%. To compensate for the increased uncertainty of the results based on the small study samples, we performed a two-step validation process; first through repeated measures of variance, and then through ANCOVA evaluation, and for mortality through Kaplan–Meier survival analyses. From these analyses, it remained clear that in the elderly Swedish population under investigation with supplementation of selenium and coenzyme Q_10_ had a significantly lower D-dimer concentration than those on placebo by preventing the increase in D-dimer concentration that seemed to take place among those on placebo.

An important issue is whether the elevated level of D-dimer in the studied cohort is a result of thromboembolism—which secondarily results in an increased level of inflammation, or whether inflammation per se resulted in an increased level of D-dimer in the absence of thrombus formation. Previous studies have indicated that an increased level of D-dimer could result from an increased non-specific inflammation without any on-going thromboembolism [54,55]. Our present results could thus be explained by the previously reported increase in inflammatory activity in elderly populations [34,35]. However, it is to be noted that even if adjusted for biomarkers of inflammation as co-variates, an independent reduction in the level of D-dimer persisted as a result of the supplementation with selenium and coenzyme Q_10_. This indicates an association between D-dimer and the supplementation beyond the role of D-dimer as a biomarker solely of inflammation.

In accordance with previous observations [56,57], we found a significant positive association between age and D-dimer in our population. Thus, the participants on placebo showed a substantial increase in the D-dimer level. This association could be a result of an increased level of inflammation as part of the normal ageing process in “healthy” subjects or could be an indicator on pathological inflammatory activity in the apparently selenium-deficient population evaluated [35]. It appears that an increase in D-dimer besides a reported positive association with inflammation, also is positively associated with endothelial dysfunction. Therefore, in those with a low baseline selenium concentration supplementation with selenium and coenzyme Q_10_ could prevent the increase in D-dimer observed in the placebo group and thereby inflammation and endothelial dysfunction, and also decrease the cardiovascular risk.

## 5. Limitations

The population analysed in this study was of a relatively small size. This increases therefore the uncertainty of the obtained results. However, as we used a two-step validation process, we argue that the results are likely to be correct. Even if the size of the study population is small, we regard the results as being interesting from a scientific point of view, and for hypothesis-generating.

The included participants represented a relatively narrow age stratum, so it is not possible to extrapolate the results to other age groups without uncertainty.

Finally, as the evaluated population consisted of Caucasians who were low in selenium and coenzyme Q_10_, it is not necessarily true that the obtained results could be extrapolated to another population.

## 6. Conclusions

D-dimer, a fragment of cleaved fibrin, reflects the fibrinolytic process, which is why it is used in clinical routines to rule out a possible thromboembolic process. However, D-dimer is also intimately related to inflammatory activity and also to endothelial dysfunction. In this report, an elderly community-living population with a relative selenium deficiency was given a dietary supplement consisting of selenium and coenzyme Q_10_. While the level of D-dimer in the placebo group increased during the intervention period, it remained unchanged or was slightly reduced in those on active treatment. After 48 months, D-dimer was significantly lower in the active treatment group in comparison with those on placebo. The results were validated through repeated measures of variance methodology and ANCOVA analyses.

We observed a significantly reduced CV mortality among those with a high D-dimer level when given selenium and coenzyme Q_10_, as compared to those on placebo. In a sub-group analysis of patients with hypertension or ischemic heart disease, the significantly lower concentration of D-dimer as a result of the supplementation could be demonstrated. High D-dimer levels in the present elderly population may reflect age-related inflammatory activity, although D-dimer may impact cardiovascular pathology beyond its role as a biomarker of inflammation.

The demonstrated results might be of interest for follow-up studies, although the present sample size was small. The results should be regarded as hypothesis-generating and it is hoped they will stimulate more research within the same area.

## Figures and Tables

**Table 1:** Baseline characteristics of the study population receiving dietary supplementation of selenium and coenzyme Q_10_ combined or placebo during four years.

	Active Treatment Group *n* = 106	Placebo Group *n* = 107	*p*-Value
Age years, mean (SD)	77.0 (3.7)	77.0 (3.3)	0.36
Gender, Males/Females	43/64	46/61	
**History**			
Diabetes, *n* (%)	25 (23.6)	21 (19.6)	0.51
Smoking, *n* (%)	9 (8.5)	12 (11.2)	0.50
Hypertension, *n* (%)	75 (70.8)	75 (70.1)	0.92
IHD, *n* (%)	18 (17.0)	22 (20.6)	0.50
NYHA class I, *n* (%)	54 (50.9)	57 (53.3)	0.73
NYHA class II, *n* (%)	33 (31.1)	26 (24.3)	0.27
NYHA class III, *n* (%)	19 (17.9)	23 (21.5)	0.51
NYHA class IV, *n* (%)	0	0	
**Medications**			
ACEI/ARB, *n* (%)	18 (17.0)	20 (18.7)	0.74
Beta blockers, *n* (%)	32 (30.2)	24 (22.4)	0.20
Diuretics, *n* (%)	29 (27.4)	32 (29.9)	0.68
Statins, *n* (%)	20 (18.9)	20 (18.7)	0.97
**Examinations**			
EF < 40%, *n* (%)	2 (1.9)	7 (6.5)	0.20
s-selenium pre-intervention µg/L, mean (SD)	67.6 (14.8)	66.3 (15.8)	0.98

Note: ACEI: ACE- inhibitors; ARB: Angiotension receptor blockers; EF: Ejection fraction; IHD: Ischemic heart disease; NYHA: New York Heart Association functional class; SD: Standard Deviation. Note: Values are means ± SDs or frequency (percent). Note: Student’s unpaired two-sided *t*-test was used for continuous variables and the chi-square test was used for analysis of one discrete variable.

**Table 2:** Analysis of covariance using D-dimer after 48 months as dependent variable.

Effects	Mean Squares	Degrees of Freedom	F	*p*
Intercept	0.24	1	6.35	0.01
Smoker	0.01	1	0.39	0.53
Hypertension	0.11	1	2.92	0.09
Diabetes	0.02	1	0.66	0.42
IHD	0.001	1	0.03	0.86
NYHA III	0.005	1	0.13	0.72
Hb < 120g/L	0.02	1	0.47	0.49
Statin treatment	0.08	1	2.16	0.15
p-selectin incl	0.002	1	0.04	0.84
hsCRP incl	0.0002	1	0.004	0.95
Endostatin incl	0.30	1	8.08	0.006
sTNF-r1 incl	0.009	1	0.23	0.63
sTNF-r2 incl	0.25	1	6.73	0.01
GDF-15 incl	0.12	1	3.14	0.08
D-dimer incl	0.95	1	25.6	0.000002
Active treatment	0.37	1	9.89	0.002
Error	0.04	1		

Note: GDF-15: Growth/differentiation factor 15; HsCRP: High sensitivity assay of CRP; IHD: Ischemic heart disease; NYHA: New York Heart Association functional class III; sTNF-r1: Tumor necrosis factor receptor 1; sTNF-r2: Tumor necrosis factor receptor 2.

## Data Availability

Under Swedish Law, the authors cannot share the data used in this study and cannot conduct any further research other than what is specified in the ethical permissions application. For inquiries about the data, researchers should first contact the owner of the database, the University of Linköping. Please contact the corresponding author with requests for and assistance with data. If the university approves the request, researchers can submit an application to the Regional Ethical Review Board for the specific research question that the researcher wants to examine.

## Abbreviations

ACEI: ACE inhibitors; ANCOVA: Analysis of covariance, ARB; Angiotension receptor blockers, CRP: C-reactive protein; CV: Cardiovascular; EF: Ejection fraction, ECG: Electrocardiogram, Hs-CRP: High sensitivity analysis of C-reactive protein, IHD: Ischaemic heart disease, NT-proBNP: N-terminal fragment of B-type natriuretic peptide; NYHA class: New York Heart Association functional class, SD: Standard deviation.
